# The development and assessment of a predicting nomogram for the recovery of immediate urinary continence following laparoscopic radical prostatectomy

**DOI:** 10.3389/fsurg.2022.1071093

**Published:** 2023-01-06

**Authors:** Zhuoran Gu, Zongtai Zheng, Wentao Zhang, Shiyu Mao, Shuai Wang, Jiang Geng, Xudong Yao

**Affiliations:** ^1^ Department of Urology, Shanghai Tenth People’s Hospital; Institute of Urinary Oncology, Tongji University School of Medicine, Shanghai, China; ^2^ Department of Radiology, Shanghai Tenth People’s Hospital, Tongji University School of Medicine, Shanghai, China

**Keywords:** prostate cancer, immediate urinary continence, laparoscopic radical prostatectomy (LRP), predictors, nomogram

## Abstract

**Purpose:**

This study aimed to develop a nomogram to predict the recovery of immediate urinary continence in laparoscopic radical prostatectomy (LRP) patients.

**Methods:**

A prediction model was developed based on a dataset of 154 LRP patients. Immediate urinary continence was defined as free from using pads within 7 days after the removal of the urinary catheter. The least absolute shrinkage and selection operator regression (LASSO) model was applied to screen the features. Multivariate logistic regression analysis was used to establish prediction model integrating the features selected from the LASSO regression analysis. Receiver operating curve (ROC), calibration and decision curve analysis (DCA) were used to assess the model's discrimination, calibration and clinical utility.

**Results:**

The identified features of the prediction model included age, body mass index (BMI) and three pelvic anatomic parameters measured by MRI: membranous urethral length (MUL), intravesical prostatic protrusion length (IPPL) and puborectalis muscle width (PMW). The nomogram showed good discrimination with an are under the curve(AUC) of 0.914 (95% CI, 0.865–0.959, *p* < 0.001). Moreover, good calibration was showed in the model. Lastly, DCA showed that the nomogram was clinically useful.

**Conclusion:**

The developed novel nomogram that can predict the possibility for post-prostatectomy patients to recover immediate urinary continence could be used as a counseling tool to explain urinary incontinence to patients after LRP.

## Introduction

Prostate cancer is currently the most common carcinoma worldwide and the mortality of it is only secondary to lung cancer (1). Radical prostatectomy (RP) is the first-line treatment option for localized prostate cancer. However, post-prostatectomy urinary incontinence, which is the leading complication of RP, significantly affects the patients' postoperative quality of life (2). It is showed that range of continence rates at 1, 3, and 12 months after RP was 33%–83.8%, 52%–92.3%, and 89%–97.9%, respectively (3). Long-term postoperative recovery of continence is similar; however, the ratio of patients who achieve early recovery of urinary continence varied considerably. The reason accounting for this diversity of continence rates at the early stage post RP were complex, but it was certain that the patients' demographics and pelvic anatomic features play important roles apart from surgical factors.

Due to the standardization of RP procedures, changes in surgical approaches, and improvement of surgical reconstruction, the recovery rate of urinary continence after RP has been reported to increase (4). Several preoperative patients' demographic characteristics, including old age (5, 6) and obesity which equals to higher body mass index (BMI) (6, 7), larger prostate volume (6, 7), and severe lower urinary tract symptom (6, 8) have been proposed to be related to post-prostatectomy urinary incontinence. Moreover, preoperative pelvic and urethral anatomic parameters measured on MRI, such as membranous urethral length (MUL) (6, 9, 10) and intravesical prostatic protrusion length (IPPL) (11, 12), have also been demonstrated to be associated with the recovery of urinary continence.

With the further recognition of periurethral and pelvic anatomy, some patients were able to recover urinary continence at an ultra-early stage after RP. This condition is called immediate urinary continence(IUC) recovery which is defined as the dependence from using pads within 7 days after RP. Numerous studies have focused on early (1–3 months after RP) or long-term recovery (12 months after RP); however, studies have tackled IUC recovery. In previous studies, it has been demonstrated that Retzius-sparing robot-assisted radical prostatectomy (RS-RARP) contributes to IUC recovery after RP by avoiding cutting off puboprostatic ligament, ligating DVC and incising Denonvilliers's fascia (13, 14). Moreover, minimal residual membranous urethral length (mRUL) (13), prostate volume and preoperative IPPS (14) have been determined as independent predictors of IUC recovery after RP. Although several predictors were reported to be related to IUC recovery, association between pelvic anatomy to this condition has not yet been thoroughly investigated. Therefore, it is meaningful to extensively measure the pelvic anatomic parameters and explore their relationship with postoperative IUC. Furthermore, based on these measurable and objective pelvic parameters, nomogram would make a difference for patients who underwent RP. Indeed, few studies have already focused on this issue. The purpose of this study was to develop a valid but simple prediction tool by measuring pelvic parameters on perioperative MRI for post-prostatectomy patients to be able to assess the possibility of recovering IUC.

## Materials and methods

### Patients

**Figure 1:**
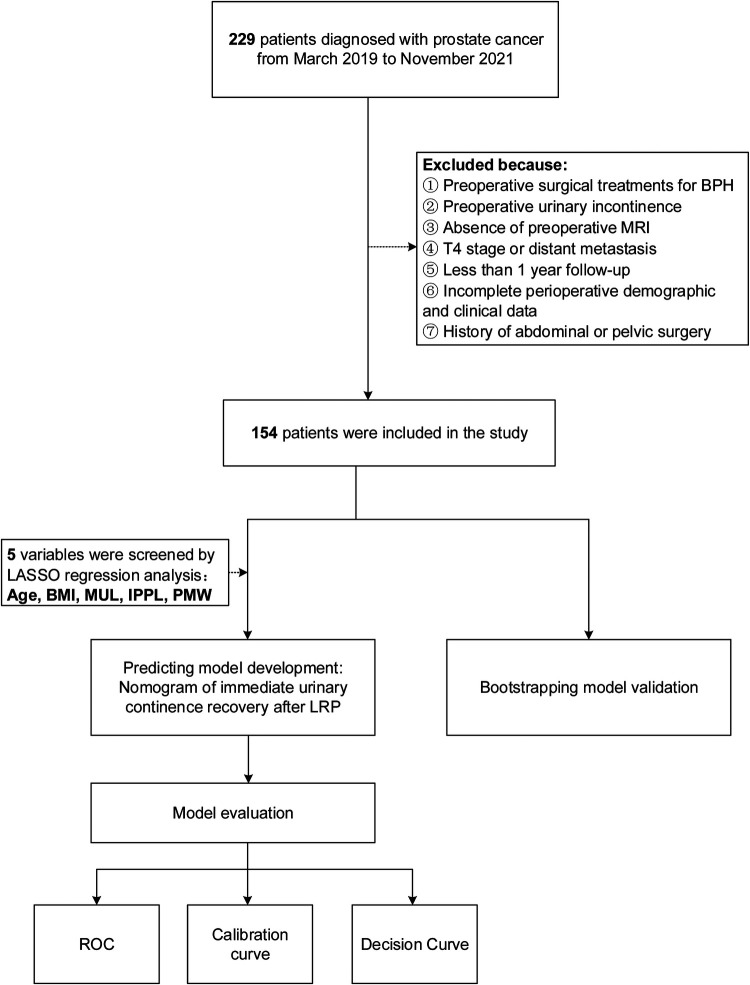
Work flowchart of the patients excluded from the study and the development of predicting model. BMI, body mass index; IPPL, Intravesical prostatic protrusion length; MUL, membranous urethral length; PMW, Puborectalis muscle width.

This study was approved by the ethics committee of Shanghai Tenth People's Hospital. A total of 229 consecutive patients with prostate cancer who underwent 3D laparoscopic radical prostatectomy(LRP) performed by the same surgeon (Dr. Xudong Yao) at Shanghai Tenth People's Hospital from March 2019 and November 2021, were reviewed retrospectively. All patients had undergone multiparameter magnetic resonance imaging (mp-MRI) to assess and measure extracapsular extension, seminal vesicle invasion, and pelvic anatomic parameters. The exclusion criteria that were used included patients with suspected lymph node invasion, or distant metastasis, or patients who had received surgical treatments for benign prostatic hyperplasia (BPH), abdominal or pelvic surgery before RP. Furthermore, patients who had preoperative urinary incontinence lacked pelvic MRI data or had missing clinical characteristics were excluded as well. After applying the exclusion criteria, 154 patients were included into the development of the prediction model based on 5 variables screened from LASSO regression model: age, BMI, MUL, IPPL, and puborectalis muscle width(PMW). Internal validation was used to validate the model, while receiver operating curve(ROC), calibration curve, and decision curve analysis(DCA) were used to assess the discrimination, calibration, and clinical usefulness of the prediction model respectively (Figure 1).

### Surgical procedure

All patients underwent extraperitoneal LRP, and neurovascular bundle preservation was not performed. The LRP procedure was performed similarly to the extraperitoneal approach described by Stolzenburg et al. (15). In brief, an extraperitoneal operating space was created using a five-spot approach. Bilateral pelvic lymphadenectomy was performed routinely. Then, the adipose tissue surrounding the prostate was resected. Afterward, the endopelvic fascia was bilaterally incised and the dorsal venous complex (DVC) was ligated with barbed thread. The bladder neck was transected backward, while the seminal vesicles were dissected from the vas deferens on both sides. Next, the Denovillier's fascia was dissected down to the apex of the prostate, with the lateral ligaments of prostate cut off. Subsequently, the distal urethra was incised and preserved when the apex was insolated.After the removal of the prostate, the posterior musculofascial was reconstructed routinely by joining the posterior median raphe with the connected dorsal wall of the external urethral sphincter(rhabdosphincter) to the residuum of the Denonvilliers's fascia and to suspend it to the posterior wall of the bladder. The procedure of posterior reconstruction is similar to the “*Rocco stich*” (16). Finally, total urethral reconstruction was performed, and the bladder neck was narrowed if necessary.

### MRI measurements

**Figure 2:**
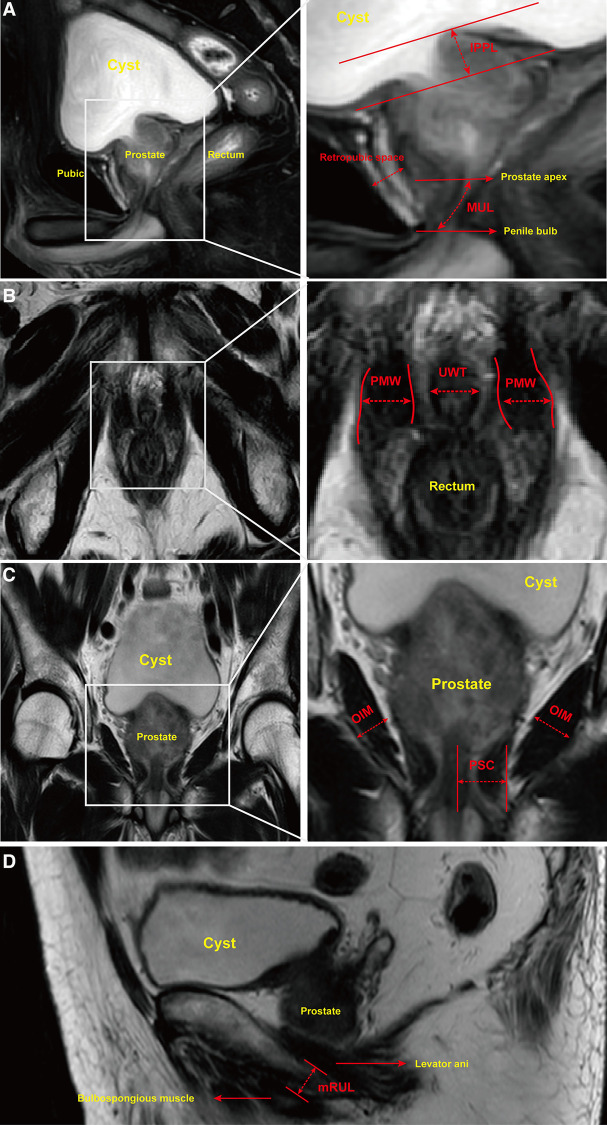
Urethral and pelvic anatomic parameters measured on MRI. Notes: (**A**) Sagittal T2-weighted sequences allowed for membranous urethral length (MUL) to be measured from inferior edge of the prostate apex to the superior margin of the penile bulb. Intravesical prostatic protrusion length (IPPL) was measured by the vertical distance from the tip of the protruding prostate to the base of bladder. Retropubic space was measured by the shortest distance between posterior margin of pubis and anterior of prostate. (**B**) Axial T2-weighted sequences allowed for puborectalis muscle width (PMW) and urethral wall thickness (UWT) to be measured. PMW was measured by the distance between inner and outer side of puborectalis muscle at the thickest portion. (**C**) Coronal T2-weighted sequences were used to measure peri-urethral sphincter complex (PSC) thickness and obturator internus muscle (OIM). (**D**) Minimal residual membranous urethral length (mRUL) was measured from the lower margin of the puborectalis muscle to the upper margin of the bulbospongiosus muscle in a direction parallel with the membranous urethra on Sagittal T2-weighted sequences.

A preoperative pelvic MRI was performed using a 3.0-T whole-body magnetic resonance scanner. Images were obtained in 2 mm slices with T2-weighted sequences of entire pelvis in the axial, sagittal and coronal views. MUL was defined as the distance from the apex of the prostate to the urethra at the level of the penile bulb in sagittal T2-weighted sequences (Figure 2A). Retropubic space was measured from posterior of pubis to anterior of prostate (Figure 2A). IPPL was measured in the sagittal T2-weighted sequences as the distance from the protruding part of the prostate to the base of the urinary bladder (Figure 2A). Axial T2-weighted sequences allowed for the urethral wall thickness (UWT) to be measured and the puborectalis muscle width (PMW) to be estimated at the thickest portion of urethral sphincter (Figure 2B). The peri-urethral sphincter complex (PSC) thickness and obturator internus muscle (OIM) were measured in coronal T2-weighted sequences (Figure 2C). Meanwhile, mRUL was measured from the lower margin of the puborectalis muscle to the upper margin of the bulbospongiosus muscle parallel with the membranous urethra on sagittal T2-weighted sequences (Figure 2D). All of the parameters discussed above were measured by one urologist and one radiologist. In case of significant differences between the two outcomes, the data of the urologist was taken as final. In measuring the parameters, the researchers were blind to the patient's postoperative urination status to eliminate the chance of introducing any bias.

### Continence evaluation and follow-up

All patients were assessed to evaluate their urination status through using on-phone questionnaires or postoperative outpatient follow-ups. IUC was defined as the independence from using pads or safety liners with one week after removing the urinary catheter. Demographic, procedural and perioperative data were collected from the hospital charts or databases.

### Construction and assessment of the nomogram

Firstly, LASSO, a method that is suitable for the reduction of high dimensional data (17, 18), was used to select the optimal predictive features of post-prostatectomy patients. Features with nonzero coefficients in the LASSO regression model were selected and incorporated to establish a prediction model using multivariate logistic regression analysis. The prediction model was performed in the form of a nomogram. Calibration curves were plotted to assess the calibration of constructed IUC nomogram. ROCs with the corresponding area under the curves (AUCs) were used to quantify the discrimination performance of the nomogram. Moreover, the nomogram was subjected to internal validation by bootstrap­ping validation (1,000 bootstrap resamples), and a *C*-index was measured. Finally, DCA was conducted to determine the clinical usefulness of the immediate urinary continence recovery nomogram by quantifying the net benefits at different threshold probabilities (19). The net benefit was calculated by subtracting the proportion of all patients who tested false positive from the proportion of the patients who tested true positive and by weighing the relative harm of forgoing interventions compared with the negative consequences of unnecessary intervention.

### Statistical analysis

Continuous normally distributed data were expressed as mean ± standard deviation (SD), while continuous non-normally distributed data were presented as the median and interquartile range (IQR). The Student's *t*-test, Mann–Whitney *U* test, Pearson *χ*^2^ test, or fisher exact test were used to determine the statistical correlation between the possibility of IUC recovery and preoperative variables. All data analyses were performed using either the SPSS 24.0 statistical software (IBM SPSS, Chicago, IL, United States) or R software (Version 4.1.1; https://www.R-project.org). In all analyses, a *p *< 0.05 was considered statistically significant and a confidence interval (CI) of 95% was assumed.

## Results

### Patients' characteristics

**Figure 3:**
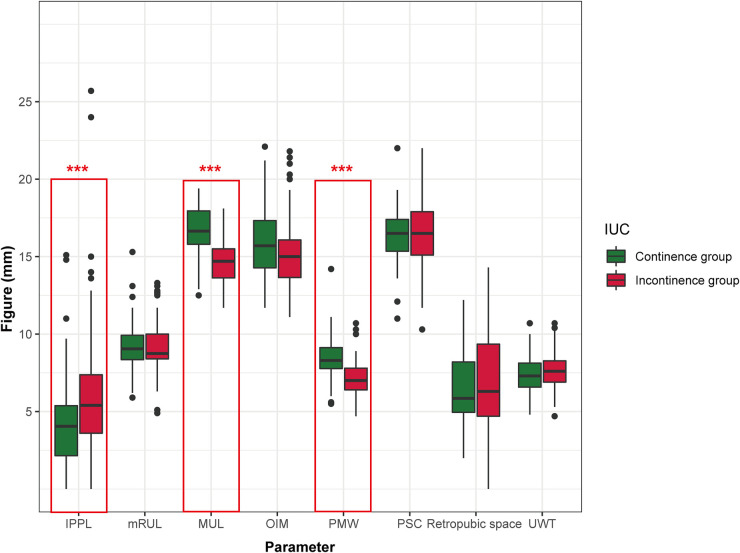
Preoperative urethral and pelvic anatomic parameters in continence vs. incontinence group immediately after LRP. IPPL, Intravesical prostatic protrusion length; MUL, membranous urethral length; PMW, Puborectalis muscle width; UWT, urethral wall thickness; PSC, peri-urethral sphincter complex; OIM, obturator internus muscle; mRUL, Minimal residual membranous urethral length. *p < 0.05; **p < 0.01; ***p < 0.001.

A total of 154 patients who underwent LRP were included in this study, and among theses, 64 (41.6%) patients were reported to have achieved the recovery of IUC. The baseline clinical characteristics and follow-up results are summarized in Table 1. Patients were stratified into two groups according to IUC status after LRP. Three pelvic anatomic parameters, including PMW (*p *< 0.001), MUL (*p *< 0.001), and IPPL (*p *= 0.003) showed significant differences between groups (Figure 3)*.* Specifically, the patients in the IUC group had significantly had longer MUL [16.65 (15.80–18.05) vs. 14.70 (13.88–18.00)], wider PMW [8.30 (7.73–9.18) vs. 7.00 (6.38–7.80)], and shorter IPPL [4.05 (2.05–5.73) vs. 5.40 (3.58–7.53)] compared to the incontinence group.

**Table 1:** Association between recovery of immediate urinary continence and perioperative characteristics.

Variables	Whole Pts	Immediate urinary continence		*P-*value
		Continence group	Incontinence group	
*N*	154	64	90	
Age (y), mean ± standard deviation	69.97 ± 6.64	68.80 ± 6.59	70.80 ± 6.58	0.065
BMI ≥ 25 (kg/m^2^), *n* (%)	60 (39.0)	22 (34.4)	38 (42.2)	0.325
Smoking history, *n* (%)	46 (29.9)	19 (29.7)	27 (30.0)	0.522
Diabetes, *n* (%)	5 (3.2)	3 (4.7)	2 (2.2)	0.697
ADT before surgery, *n* (%)	15 (9.7)	5 (7.8)	10 (11.1)	0.496
PSA (ng/ml), median (interquartile range)	12.05 (7.02,22.0)	13.5 (8.10,28.90)	11.08 (6.97,20.00)	0.140
Operation time (min), median (interquartile range)	160 (125.00,180.00)	170 (131.25,185.00)	155 (120.00,176.25)	0.130
Estimated blood loss (ml), median (interquartile range)	100 (50,200)	100 (50,200)	100 (50,200)	0.756
Drainage removal time (d), median (interquartile range)	6 (5,10)	6 (5,10)	7 (5, 11)	0.448
Postoperative length of stay (d), median (interquartile range)	9 (6,12)	9 (6,12)	9 (7,12.25)	0.435
Gleason score, *n* (%)				0.480
≤6	3 (1.9)	1 (1.6)	2 (2.2)	
7	98 (63.6)	39 (60.9)	59 (65.6)	
≥8	53 (34.4)	24 (37.5)	29 (32.2)	
Pathological T stage				0.824
≤T2	87 (56.5)	35 (54.7)	52 (57.8)	
T3a	30 (19.5)	12 (18.8)	18 (20)	
≥T3b	37 (24)	17 (26.6)	20 (22.2)	
Lymph node metastasis, *n* (%)	7 (4.5)	3 (4.7)	4 (4.4)	1.000
Risk group^a^, *n* (%)				0.620
Low	2 (1.3)	1 (1.6)	1 (1.1)	
Intermediate	6 (3.9)	3 (4.7)	3 (3.3)	
High	146 (94.8)	60 (93.8)	86 (95.6)	
International IPSS, *n* (%)				0.382
Mild	119 (77.3)	52 (81.3)	67 (74.4)	
Moderate	27 (17.5)	8 (12.5)	19 (21.1)	
Severe	8 (5.2)	4 (6.3)	4 (4.4)	
Prostate volume (ml), median (interquartile range)	38.22 (29.23,49.85)	38.22 (28.38,49.88)	38.10 (28.85,50.00)	0.852
PMW (mm), median (interquartile range)	7.60 (6.60,8.40)	8.30 (7.73,9.18)	7.00 (6.38, 7.80)	<0.001***
MUL (mm), median (interquartile range)	15.35 (14.28,16.73)	16.65 (15.80, 18.05)	14.70 (13.58,15.50)	<0.001***
IPPL (mm), median (interquartile range)	4.70 (3.23, 6.90)	4.05 (2.05,5.73)	5.40 (3.58,7.53)	0.003**
OIM (mm), median (interquartile range)	15.10 (13.80,16.60)	15.70 (14.23,17.40)	15.00 (13.60,16.13)	0.080
PSC (mm), median (interquartile range)	16.50 (15.10,17.90)	16.50 (15.25,17.40)	16.50 (15.10,17.90)	0.762
UWT (mm), median (interquartile range)	7.50 (6.60,8.23)	7.30 (6.53,8.18)	7.60 (6.88,8.33)	0.147
mRUL (mm), median (interquartile range)	8.90 (8.40,10.00)	9.05 (8.25,9.98)	8.75 (8.40,10.00)	0.933
Retzius’ space (mm), median (interquartile range)	6.00 (4.70,8.65)	5.85 (4.85,8.20)	6.30 (4.58,9.40)	0.637
Extracapsular extension, *n* (%)	67 (43.5)	29 (45.3)	38 (42.2)	0.703
Seminal vesicle invasion, *n* (%)	37 (24.0)	17 (26.6)	20 (22.2)	0.534

BMI, body mass index; PSA, prostate specific antigen; IPSS, International prostate symptom score; IPPL, Intravesical prostatic protrusion length; MUL, membranous urethral length; PMW, Puborectalis muscle width; OIM, Obturator internus muscle; PSC, Peri-urethral sphincter complex thickness; UWT, Urethral wall thickness; mRUL, minimal residual membranous urethral length.

^a^ D’Amico classification.

* *p* < 0.05; **p < 0.01; ***p < 0.001.

### Feature selection

**Figure 4:**
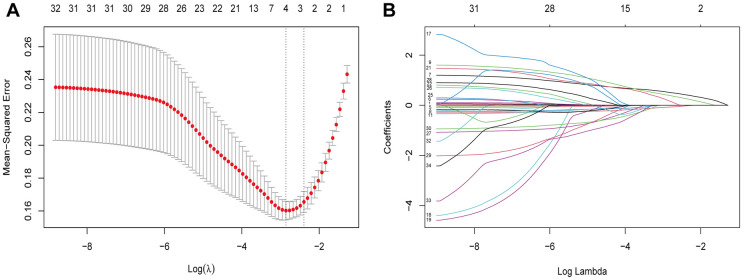
Demographic and clinical features selection using the LASSO regression model. Notes: (**A**) Optimal parameter (lambda) selection in the LASSO model used fivefold cross-validation *via* minimum criteria. The partial likelihood deviance (binomial deviance) curve was plotted vs. log(lambda). Dotted vertical lines were drawn at the optimal values by using the minimum criteria and the 1 SE of the minimum criteria (the 1-SE criteria). (**B**) LASSO coefficient profiles of the 34 features. A coefficient profile plot was produced against the log(lambda) sequence. Vertical line was drawn at the value selected using fivefold cross-validation, where optimal lambda resulted in five features with nonzero coefficients. LASSO, least absolute shrinkage and selection operator; SE, standard error.

Based on the demographic and, perioperative features of 154 patients in the cohort, 34 candidate features were reduced to five potential predictors with nonzero coefficients in the LASSO regression model (Figures 4A,B). The final features included age, BMI, MUL, PMW, and IPPL (Table 2).

**Table 2:** Prediction factors for the recovery of immediate urinary continence with patients following LRP.

Variables	Prediction model				
	*β*	OR	95%CI		*P*
BMI ≥ 25 kg/cm^2^	−1.150	0.317	0.105	0.863	0.031*
Age (y)	−0.033	0.967	0.899	1.039	0.361
IPPL (mm)	−0.272	0.762	0.645	0.875	<0.001**
MUL (mm)	0.947	2.577	1.824	3.878	<0.001**
PMW (mm)	0.917	2.503	1.634	4.059	<0.001**

OR, odds ratio; CI, confidence interval; BMI, body mass index; IPPL, Intravesical prostatic protrusion length; MUL, membranous urethral length; PMW, Puborectalis muscle width.

*β* is the regression coefficient; *p < 0.05; **p < 0.001.

### Development of the prediction model

**Figure 5:**
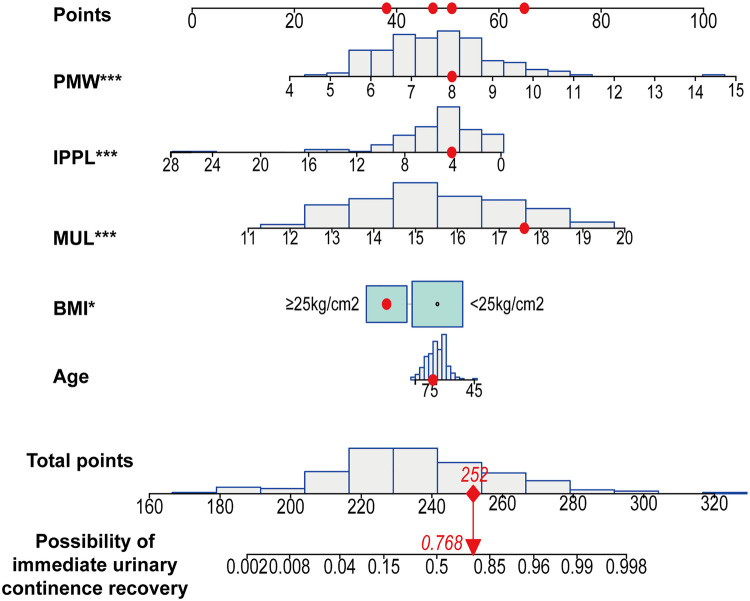
Developed immediate urinary continence recovery nomogram. Note: The immediate urinary continence recovery nomogram was developed in the cohort, with age, body mass index (BMI), the membranous urethral length (MUL), puborectalis muscle width (PMW) and the intravesical prostatic protrusion length (IPPL) were incorporated. *p < 0.05; **p < 0.01; ***p < 0.001. BMI, body mass index; IPPL, Intravesical prostatic protrusion length; MUL, membranous urethral length; PMW, Puborectalis muscle width.

Based on the multivariate regression analysis, BMI ≥ 25 kg/m^2^ (OR, 0.317; 95%CI, 0.105–0.863; *p *= 0.031), MUL (OR, 2.577; 95%CI, 1.824–3.878; *p *< 0.001), IPPL (OR, 0.762; 95%CI, 0.645–0.875; *p *< 0.001) and PMW (OR, 2.503; 95%CI, 1.634–4.059; *p *< 0.001) were significant predictors of IUC after LRP (Table 2). A model that incorporated these independent predictors, as well as age (OR, 0.967; 95%CI, 0.899–1.039, *p *= 0.361) was used to construct the nomogram (Figure 5).

### Apparent performance of the IUC recovery nomogram

**Figure 6:**
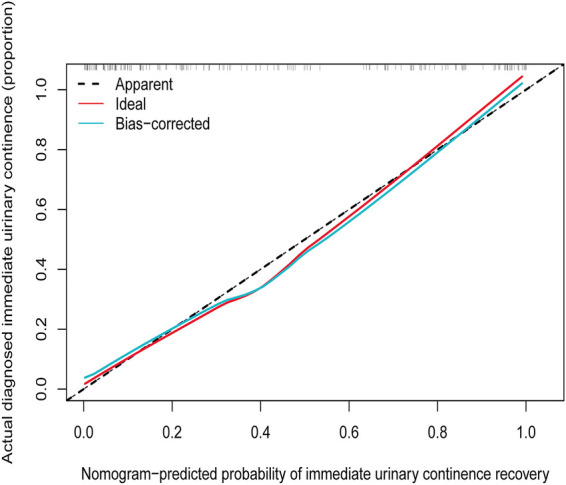
Calibration curves of the immediate urinary continence recovery nomogram prediction in the cohort. Notes: The *x*-axis represents the predicted possibility of immediate urinary continence recovery. The *y*-axis represents the actual diagnosed immediate urinary continence. The diagonal dotted line represents a perfect prediction by an ideal model. The solid line represents the performance of the nomogram, of which a closer fit to the diagonal dotted line represents a better prediction.

**Figure 7:**
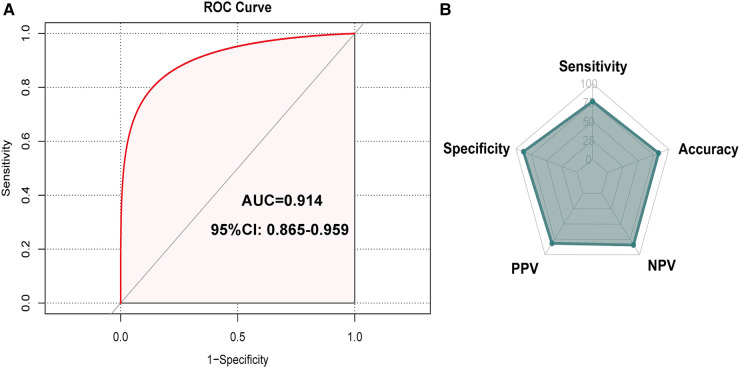
Receiver operating curve (ROC) that showed performance of predicting model. Note: the cut-off level of >69.0 nomogram predicted immediate urinary continence recovery (sensitivity = 76.6%, specificity = 87.8%, positive predictive value = 81.7%, negative predictive value = 84.0%, accuracy = 83.1%), and an area under curve (AUC) of 0.914 (95% CI: 0.865-0.959, *p* < 0.001). AUC, area under the curve.

The *Hosmer*-Lemeshow test (*Hosmer*-Lemeshow statistic ≥0.05) was used to evaluate the model's goodness of fit and was presented as the calibration curve, while the ROC analysis was used to assess the discrimination of the model, and. The calibration curve of the nomogram for the prediction of IUC recovery in post-prostatectomy patients demonstrated good agreement in this cohort (Figure 6). In the ROC analysis, with a cut-off level of >69.0, the nomogram was able to predict IUC recovery (sensitivity = 76.6%, specificity = 87.8%, positive predictive value = 81.7%, negative predictive value = 84.0%, accuracy = 83.1%), and with AUC of 0.914 (95%CI: 0.865–0.959, *p *< 0.001); thus, showing, good discrimination (Figure 7). A high *C*-index value of 0.900 was reached in the internal validation as well. Overall, the IUC nomogram's apparent performance addressed a good prediction capability.

### Clinical application

**Figure 8:**
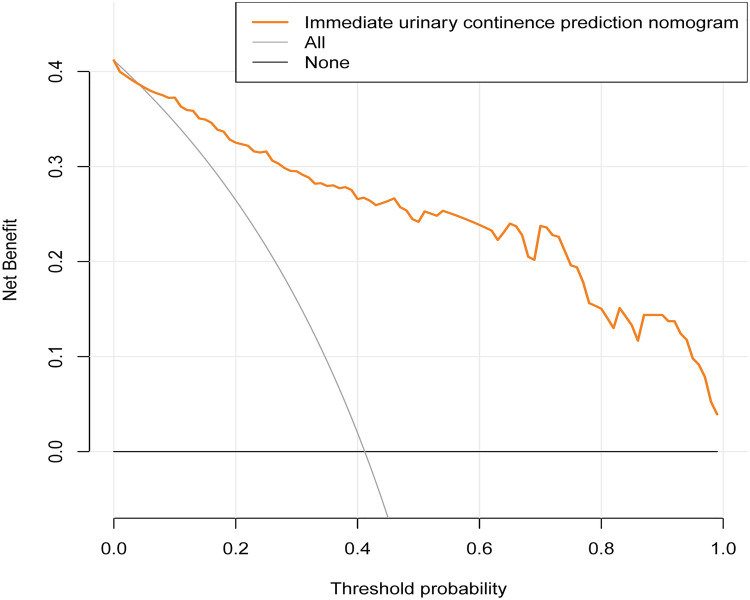
Decision curve analysis for the immediate urinary continence recovery nomogram. Notes: The *y*-axis measures the net benefit. The dotted line represents the immediate urinary continence nomogram. The thin solid line represents the assumption that all patients achieving the recovery of immediate urinary continence. Thin thick solid line represents the assumption that no patients are immediate urinary continence. The decision curve showed that if the threshold probability of a patient is 1% using this nomogram in the current study to predict immediate urinary continence possibility adds more benefit than the intervention-all-patients scheme or the intervention-none scheme.

The DCA showed that in case where the threshold probability of a patient is >1%, using this nomogram to predict the recovery of IUC in patients following LRP adds more benefit (Figure 8). Within this range, the net benefit of the IUC nomogram was comparable, with several overlaps.

## Discussion

Numerous studies demonstrate that nomograms that incorporate multiple predictive elements outperform the prognosis prediction that is only based on specialists' experience or other simpler models (20, 21). The development and utility of our nomogram can distinguish the post-prostatectomy patients who could reach the immediate recovery of urinary continence from those who experience delayed recovery. Furthermore, it gives opportunities for both patients and urologists to better understand the occurrence of constant incontinence and offer the appropriate medical intervention as soon as possible. Nomograms are currently widely used in predicting the specific survival of PCa patients. Similarly, albeit in limited amounts, several nomograms are available to predict the functional outcomes of patients following LRP, such as predicting the immediate recovery of urinary continence post-surgery. To the best of our knowledge, only five nomograms constructed for predicting the recovery of urinary continence (5, 22–25). However, none of these nomograms have focused on the immediate recovery of urinary continence after RP. Besides, most of these nomograms lack important and novel pelvic anatomic parameters, which can be easily measured on MRI and are directly associated with the immediate recovery of urinary continence after RP. Therefore, it is meaningful to develop a reasonably accurate post-LRP nomogram for predicting IUC recovery to guide counseling and follow-up for PCa patients, as well as to help direct urologists to pay attention to the pelvic parameters incorporated in the model.

We developed a novel prediction tool for the recovery of IUC among post-prostatectomy patients. This nomogram which incorporated patients' age, BMI and three pelvic anatomic parameters including MUL, PMW and IPPL, demonstrated good discrimination performance and calibration. This constructed nomogram suggested that age, BMI, MUL, PMW, and IPPL were critical individual features that determine whether patients after RP were able to recover IUC.

Stress urinary incontinence (SUI) is the main type of urinary incontinence after RP. According to urodynamics, keeping the urethral closure pressure higher than the intravesical pressure is crucial for maintaining the function of urinary continence. The urethral closure pressure consists of forces contributed by the rhabdosphincter (external urethral sphincter), internal urethral sphincter, prostatic and membranous urethra, and periurethral supportive structures, including theanterior, posterior supportive structures and pelvic floor tissue. The anterior supportive structures include the pubo-urethral, pubovesical, and puboprostatic ligament, and the tendinous arch of endopelvic fascia. Meanwhile, the posterior supportive structures include the perineal body and Denonvillier's fascia. The pelvic floor tissue consists of the levator muscle complex and peripheral fascia. Unfortunately, RP causes damage to the internal and external urethral sphincter, and periurethral supportive structures, which subsequently leads to a decrease in the urethral closure pressure (26). Moreover, RP causes lesions in the bladder neck and interruption of detrusor muscle continuity, which the leads to detrusor instability and decreased compliance of bladder (27). Taken together, the pressure differences between the urethra and bladder are unsustainable due to surgical injuries, especially the injury in the sphincter.

The membranous urethra is defined as the part of the urethra between the apex of the prostate and the urethral bulb. This structure is mainly composed of smooth muscle fibers, and its full length is surrounded by an external urethral muscle which is formed following an omega-shaped. The entire membranous urethra coordinates with the external urethral muscle to maintain the urethral closure pressure, which plays an important role in the recovery of urinary continence after RP. Unfortunately, different approaches of RP cause the lesion or loss of membranous urethra and external urethral muscle, leading to a decrease in urethral pressure, and finally causing urinary incontinence. The MUL can be practically considered as the length of the external urethral muscle. Therefore, a longer membranous urethra means a greater amount of smooth muscle fibers and external urethral muscle, which both contributes to achieving immediate urinary continence recovery.

In a previous study, MUL was demonstrated as an important predictor of urinary continence after RP (28, 29). A meta-analysis consisting of 13 studies proved that patients with longer preoperative MUL spend less time achieving post-prostatectomy urinary continence and suggested to predict the recovery of urinary ontinence after RP by measuring MUL in T2 sequences of preoperative pelvic MRI (10). Consistent with theses, our study also revealed that MUL plays an important role in the recovery of IUC after RP.

On the basis of MUL, Satake et al. proposed the concept of the minimal residual membranous urethral length (mRUL), which is defined as the distance between the lower margin of the puborectalis muscle to the upper margin of the bulbospongiosus muscle in a direction parallel with the urethra, and representing the minimal intact residual part of the membranous urethra during RP (28). Satake at el. suggested that mRUL is measured as a distal part of the urethra, away from the surgical field, and is generally preserved from operative damage during RP. Furthermore, the and border of both sides of the mRUL is more clear on MRI compared with MUL. In this retrospective cohort study which included 113 PCa patients, the mRUL was proved as an independent predictor of urinary continence recovery after RP (28). Moreover, Qiu et al*.* retrospectively studied 110 PCa patients following RS-RARP and suggested that mRUL was an independent predictor of urinary continence recovery as well (13).

However, our study did not show any significant different in the mRUL of the IUC and incontinence groups. Moreover, there are few prospective, muti-center, and large samples studies to further support that mRUL is a better predictor of urinary continence recovery than MUL. Overall, MUL is still the currently reliable predictor of urinary continence recovery after RP.

A study tried to determine the association between the levator ani muscle and recovery of urinary continence. Song at el. proposed that the size ratio of the levator ani muscle to prostate volume is associated with early recovery of urinary continence after RP (30). However, another study revealed that the anatomically close relation between the levator ani muscle and membranous urethra are independent predictor of continence recovery after RP (31). The levator ani muscle has three components: puborectalis, pubococcygeus and iliococcygeus muscles. It is the puborectalis muscle that is closely related to the urethra rather than the other two parts. Besides, the actual measurement of the puborectalis muscle on MRI had not been standardized. In our study, we measured the widest portion of the puborectalis muscle as a parameter and found that PMW was an independent predictor of IUC recovery after RP. To the best of our knowledge, our study is the first time to reveal that PMW is an objective and quantitative factor for predicting the recovery of IUC after RP. Although it is unclear how PMW functions in the recovery of IUC, we proposed a hypothesis that due to the surgical damage incurred by the supportive structures surrounding the urethra and loss of membranous urethra, the puborectalis muscle assists the external urethral sphincter to maintain the urethral closure pressure, especially in an ultra-early period after RP. As an important part of the pelvic floor muscle, the puborectalis muscle may add an extra closure force for the membranous urethra to increase the urethra closure pressure by directly squeezing the urethra or increasing the intrapelvic pressure.

Other preoperative pelvic parameters are related to urinary continence in previous studies. In our study, preoperative IPPL was demonstrated to be an important predictor of urinary continence recovery after RP (11). Severe intravesical prostatic protrusion leads to bladder outlet obstruction and bladder dysfunction, which may delay the recovery of urinary continence after RP. Besides, longer IPPL forces urological surgeons to cause more damage to the bladder neck to remove the entire prostate and keep the surgical margin negative, which means more loss of internal sphincter muscle and disruption of bladder compliance. Consequently, the intravesical pressure increases, but the urethral closure pressure decreases, leading to urinary incontinence after RP. Several studies found that complete bladder neck preservation (32, 33) and reconstruction (34, 35) could promote urinary continence recovery, which also proved that IPPL delayed the post-prostatectomy urinary continence by influencing the dissection or preservation of the bladder neck. In our study, patients of IUC group had shorter IPPL. Furthermore, IPPL served as a risk factor for IUC.

In addition to pelvic parameters, a high BMI classification (BMI ≥ 25 kg/m^2^) was also presented as an independent predictor of IUC as well. Similar to our study, *Wolin* et al*.* suggested that obese subjects had a higher incontinence rate after RP than normal subjects and patients who were overweight and lacked physical activity were more likely to delay the recovery of urinary continence (7). However, other studies believed that BMI did not affect the risk of postoperative urinary incontinence (6). Therefore, the effect of BMI on urinary continence recovery after RP is still unclear and needs further investigation.

The range of continence rates at 12 months after RP was 89%–97.9%, which was similar and stable (3). The factors mainly affecting long-term urinary continence included age, BMI, prostate volume and MUL (6). Long-term urinary continence after RP was fully investigated in previous studies, but there were few studies focusing on the IUC recovery. The range of continence rates at immediate or early stage after RP was 33%–83.8%, which varied considerably (3). RS-RARP and NVB-sparing technique (13), mRUL (13), prostate volume and preoperative IPPS (13, 14) were proposed to affect the IUC recovery. Therefore, there were common factors affecting both IUC and long-term urinary continence, including age, BMI, and MUL. Due to less information regarding IUC, more investigation should be done to reveal the relationship between IUC and long-term urinary continence.

In addition to patient-related factors, pelvic floor reconstruction aims to recover the normal pelvic anatomic relationships and preserve the periurethral supportive structures to improve the recovery of continence. The posterior reconstruction(“*Rocco*” stitch) was one of common reconstruction methods and was routinely performed among our patients. Posterior reconstruction was able to reduce the tension of vesicourethral anastomosis by realignment of the tissues dorsal to the bladder and the urethra (16). A meta-analysis suggested that posterior reconstruction improved the early recovery of urinary continence after RP (36). However, the only two randomized controlled trials (RCTs) included in this meta-analysis instead demonstrated that patients undergoing posterior reconstruction had no significant improvement on the early recovery of urinary continence (37, 38). Therefore, it currently lacked solid evidence that posterior reconstruction contributed to the recovery of urinary continence.

Besides, perioperative nursing specially the pelvic floor muscle exercise (PFME) was encouraged among the patients before and after RP. Previous studies supported that preoperative PFME improved the post-prostatectomy urinary continence recovery at 3 months after RP (39). How PFME improves the recovery of urinary continence is still unclear, but it is confirmable that PFME could enhance the efficiency and force of levator ani muscle contraction. Therefore, we could reasonably suppose that as the important components of periurethral supportive structures, pelvic floor muscles coordinating with other reserved anatomic structures during RP promote the early recovery of urinary continence, which further certifies PMW's critical role in the IUC recovery. However, few solid evidence demonstrated that postoperative PFME could add benefit in the urinary continence recovery (40). Duration of PFME may explain the different effectiveness between pre- and post-prostatectomy PFME. Compared with postoperative PFME, PFME before RP allows patients to strengthen their pelvic floor muscles with a longer time and get used to controlling their pelvic floor muscle, which contributes to the early recovery of urinary continence. Overall, PFME is encouraged to be performed before RP and is beneficial to urinary continence recovery. The effect of postoperative PFME on urinary continence recovery needs further validation.

This study had several limitations that need to be considered. Firstly, the patients included in the study underwent the extrafascial RP without preserving the neurovascular bundle (NVB) during RP. Intrafascial RP (NVB-sparing RP) improves the early recovery of urinary continence (41), but was only applicable to the PCa patients with tumor confined within prostate (T stage ≤T2c). In our study, there were a large number of elderly patients (average age is 69.97), and 43.5% of patients were extracapsular extensive, and only eight (2 plus 6 equals 8) patients were low-intermediate risk (Table 1), which meant a lot of patients were not applicable for preserving the NVB. Besides, NVB is fine anatomic structure and vulnerable to surgical damage. The intraoperative neuro-electrophysiologic tests can accurately assist surgeon to identify the NVB during RP and assess its electrophysiologic status after RP (42). Nevertheless, the identification of NVB and electrophysiologic assessment of NVB commonly depend on surgeon's experience. Although intrafascial RP was used to preserve NVB, imperceptible surgical injury could cause damage to NVB and affect its function. Therefore, it is difficult to confirm whether the NVB was anatomically and functionally preserved. Secondly, all the operations were LRP and performed by one surgeon who was the chief urologist in our institution. Although this can reduce the differences in the IUC by different surgeons' preference and different surgical approaches, the applicability of the nomogram is meanwhile influenced. Lastly, although our nomogram was robustly examined with internal validation by bootstrap testing, external validation should be done in a wider post-prostatectomy patient population from other institutions. It is of better applicability and accuracy for the nomogram to include the patients following RP in different approaches including open RP and RARP, and NVB-sparing RP. Nonetheless, we believed that our study was able to reflect the importance of PMW, MUL, and IPPL in the IUC recovery after LRP.

## Conclusion

This study developed a novel nomogram with relatively good accuracy to help urologists estimate the probability of recovering of IUC in post-prostatectomy patients before surgery. Using the estimate of individual probability estimates, surgeons and patients can take more appropriate measures for urination monitoring and medical interventions.

## Data Availability

The original contributions presented in the study are included in the article/Supplementary Material, further inquiries can be directed to the corresponding author/s.
